# Circular RNA circEMB promotes osteosarcoma progression and metastasis by sponging miR-3184-5p and regulating EGFR expression

**DOI:** 10.1186/s40364-022-00442-9

**Published:** 2023-01-07

**Authors:** Jianye Tan, Bingsheng Yang, Haobo Zhong, Mengliang Luo, Zexin Su, Chao Xie, Meiling Shi, Chunhan Sun, Lijun Lin

**Affiliations:** 1 Department of Joint and Orthopedics, Zhujiang Hospital, Southern Medical University, Guangzhou, 510282 China; 2 Department of Orthopaedics, The Second Affiliated Hospital of Nanchang University, Nanchang, 330006 China; 3 Department of Orthopaedics, Guangdong Provincial Key Laboratory of Bone and Cartilage Regenerative Medicine, Nanfang Hospital, Southern Medical University, Guangzhou, 510515 China; 4 Department of Orthopaedic, Huizhou First Hospital, Guangdong 516003 Huizhou, China; 5 Department of Rheumatology and Clinical Immunology, Jiangxi Provincial Peoples’ Hospital Affiliated to Nanchang University, Nanchang, 330006 China

**Keywords:** Osteosarcoma, CircEMB, miR-3184-5p, EGFR, Chemoresistance

## Abstract

**Background:**

Osteosarcoma (OSA) is the most prevalent type of bone cancer with a high rate of metastasis. Circular RNAs (CircRNAs) play an essential role in multiple aspects of tumour biology. This study aimed to elucidate the role of circEMB in OSA.

**Methods:**

circRNAs related to OSA invasion were identified via RNA sequencing and qRT-PCR. The relationship between circEMB levels and clinicopathological features of OSA was examined using the clinical specimens and data of 53 patients with OSA. Several in vivo and in vitro experiments, including intravital imaging, whole-transcriptome sequencing, transwell assay, flow cytometry, dual-luciferase reporter assay, RIP assay, RNA pull-down assay and RNA-FISH, were performed to examine the effects of circEMB on the malignant behaviour of OSA.

**Results:**

A novel circRNA, named circEMB (hsa_circ_001310), was identified in this study. circEMB can promote the malignant behaviour of OSA. In vitro experiments revealed that circEMB knockdown decreased cell proliferation, inhibited tumour invasion and metastasis; increased apoptosis and resulted in G1/S phase arrest. In vivo experiments revealed that circEMB knockdown inhibited tumour growth and metastasis in xenograft-bearing mice. Mechanistically, circEMB affects the malignant behaviour of OSA by mediating EGFR as an miR-3184-5p sponge. In addition, the circEMB/miR-3184-5p/EGFR axis modulates methotrexate (MTX) resistance in OSA.

**Conclusions:**

CircEMB plays a critical role in promoting cancer via the miR-3184-5p/EGFR pathway, indicating that circEMB may serve as a therapeutic target for OSA.

**Supplementary Information:**

The online version contains supplementary material available at 10.1186/s40364-022-00442-9.

## Introduction

Osteosarcoma (OSA) is one of the most prevalent types of bone cancer, which often originates in the metaphysis of the extremities such as the distal femur and proximal tibia [1, 2]. Owing to its high degree of invasiveness, approximately 10–15% of newly diagnosed patients with OSA have metastatic disease, which reduces the 5-year survival rate from 60 to 20% and significantly affects the clinical prognosis of patients [3–5]. At present, therapeutic options for OSA include surgery and neoadjuvant chemotherapy. However, the survival rate of patients with metastasis and resistance to chemotherapy is relatively low [6]. Numerous studies have reported that gene variants play a crucial role in the pathogenesis and prognosis of OSA [7, 8]. Therefore, molecular mechanisms underlying the pathogenesis and development of OSA should be elucidated, and specific therapeutic targets should be identified.

Circular RNAs (circRNAs) are a new type of endogenous RNA. They form a continuous covalent closed-loop structure via reverse splicing of precursor mRNA (pre-mRNA) transcripts and lack the 5′-cap structure and 3′-poly-A tail [9–11]. circRNAs are highly stable and conserved because they are resistant to exonuclease activity [12]. Therefore, they can be used as novel biomarkers for diseases. circRNAs contribute to the occurrence and development of OSA by controlling the proliferation, invasion and metastatic abilities; apoptosis; metabolism and drug resistance of tumour cells [13–15]. Therefore, understanding the functions of circRNAs is important for elucidating molecular mechanisms underlying the development of OSA and for overcoming chemotherapy resistance.

In the present study, we identified a novel circRNA named circEMB (circBase ID: hsa_circ_001310 or hsa_circ_0001481) via high-throughput sequencing analysis of tissues specimens collected from patients with OSA [16]. circEMB is formed via the splicing of exons 3–9 of the parent gene EMB (aliases: Embigin) [17, 18]. The results revealed that circEMB was upregulated in OSA tissues compared with normal tissues, and its increased expression was associated with the proliferation, invasion and metastatic abilities and apoptosis of OSA cells. Mechanistically, circEMB can affect the expression of epidermal growth factor receptor (EGFR) by regulating the sponging activity of miR-3184-5p, and the circEMB/miR-3184-5p/EGFR axis can regulate methotrexate (MTX) resistance.

## Materials and methods

### Cell lines

The human normal osteoblast cell line hFOB1.19 and human OSA cell lines 143B, U2OS, MNNG, MG63 and Saos-2 were obtained from the American Type Culture Collection (ATCC, Manassas, VA). The cells were cultured in Dulbecco’s Modified Eagle’s medium (DMEM) (Gibco, USA) supplemented with 10% fetal bovine serum (BI, Israel), 1% penicillin G and streptomycin (Gibco, USA). All OSA cell lines were cultured in an incubator with 5% CO_2_ at 37 °C, whereas hFOB1.19 cells were cultured at 34 °C and 5% CO_2_. The MTX-resistant OSA cell line U2OS was provided by Dr. M. Serra (Istituti Ortopedici Rizzoli, Bologna, Italy). U2OS cells were continuously cultured in a medium containing 300-ng/mL MTX.

### RNA sequencing

RNA sequencing was performed and libraries were constructed by Gene Denovo Biotechnology (Guangzhou, China). The samples were processed and sequenced on an Illumina HiSeqTM 2500 platform. The offline data obtained were filtered and compared with the reference genome to obtain the gene expression data, which were compared in the RPM format. Ensembl_release98 served as the reference genome [19]. RNA sequencing of circRNAs was performed after the removal of conventional ribosomal RNA and degradation of linear RNA with the RNase R enzyme. The obtained circRNAs were fragmented in a fragmentation buffer, and a library was subsequently constructed.

### Real-time reverse transcription polymerase chain reaction (qRT-PCR)

Total RNA was extracted from whole cell lysates of paracancerous tissues, cancerous tissues and cells from patients with OSA using the RNAiso Plus kit (TaKaRa, Japan) according to the manufacturer’s instructions. The quality of the extracted RNA was determined on the NanoDrop 2000 spectrophotometer. Two transcription reagents were used to convert mRNA, circRNA and miRNA to cDNA. Except for miRNA, for which we used Evo M-MLV RT Kit (Accurate Biotechnology, China), we use PrimeScript RT Reagent Kit (TaKaRa, Japan). We used two reagents to perform real-time amplification for circRNA and miRNA, namely SYBR Green Premix Pro Taq HS qPCR Kit (Accurate Biotechnology, China) and SYBR Premix Ex Taq II (TaKaRa, Japan). All qRT-PCR reactions were performed utilizing the Bio-Rad CFX connect system (Bio-Rad, CA, USA). The specific program was designed as previously described [20]. The relative gene expression was determined using the 2 –^ΔΔCT^ method. The specific primers are listed in the Additional file 1: Table S1.

### Fluorescence in situ hybridization (FISH)

FISH assay was conducted for detection of the location of circEMB and miR-3184-5p in OSA cells. Cy3-labeled circEMB and FAM-labeled miR-3184-5p probes were used to detect the localization of circEMB and miR-3184-5p in OSA cells, respectively. According to the manufacturer’s instructions, the cells were inoculated in confocal dishes and fixed using 4% paraformaldehyde. After that, the cell was incubated with oligo probe overnight at 37 °C. Finally, samples were acquired by confocal microscopy (AX confocal, Nikon, Tokyo, Japan) to obtain images.

### RNA interference (RNAi)

For circEMB knockdown, two shRNAs targeting the back-splice junction of circEMB were generated by IGE Co., Ltd. (Guangzhou, China). Then, shRNA against circEMB and shRNA-NC as negative control were packaged into lentiviruses by Genechem Co., Ltd. (Shanghai, China). The miR-3184-5p mimic/inhibitor, and their corresponding negative controls (NC) were purchased from IGE Co., Ltd. (Guangzhou, China). The lentivirus-miR-3184-5p inhibitor and lentivirus-sh-EGFR were also procured from Genechem Co., Ltd. (Shanghai, China). All vectors were validated via sequencing. Lentivirus infection was performed using the HiTransG reagent (Genechem, China), as per the manufacturer’s specifications.

### Cell proliferation assay

The proliferation of OSA cells was detected using the cell counting kit-8 (CCK-8, GLPBIO, USA). Cells in different groups were quantified using a cell counting method. Subsequently, 2000 OSA cells (100 uL) in the logarithmic phase were seeded in a 96-well plate, and phosphate-buffer saline (PBS) was introduced into the marginal well. The growth and proliferation of cells were detected after 6, 24, 48 and 72 h by CCK-8 assay. A total of 10 uL of CCK-8 detection solution was introduced into every well and subjected to incubation at 37 °C for 2 h.

### Colony formation assay

A total of 800 OSA cells were seeded in a 6-well plate, and the medium was changed every 2 days. After 14 days of culture at 37 °C, cell colonies were stained with 4% polymethanol and 0.1% crystal violet and counted.

### Migration and invasion assays

transwell chamber was used to assess the migration and invasion abilities of OSA cells. Transfected OSA cells were re-suspended in 200 uL of DMEM and added to the upper compartment, whereas a medium containing 10% FBS was added to the lower compartment. The transwell chamber was incubated at 37 °C for 24 h. Thereafter, cells in the upper compartment were wiped using cotton swabs, whereas those in the lower compartment were fixed with paraformaldehyde, stained with crystal violet and counted. For invasion assay, the upper compartment was precoated with 50 uL of Matrigel (BD Bioscience, USA) to detect intrusion.

### Wound healing assays

OSA cells were seeded in 6-well plates, and a scratch was created in the cell monolayer with a 200 μL pipette tip after 90% confluency was achieved. After the scratch was created, the cells were cultured in a serum-free medium and photographed at 0 and 24 h.

### Flow cytometry

Flow cytometry was used to assess apoptosis and cell cycle progression. Cells in the logarithmic growth phase were digested in trypsin without ethylenediaminetetraacetic acid (EDTA) and washed thrice with PBS. Apoptosis was determined using the Annexin V-APC Apoptosis Assay kit (BestBio, China) according to the manufacturer’s instructions. For cell cycle analysis, cells were lysed in trypsin containing EDTA, washed thrice with PBS and fixed with ethanol overnight. Cell cycle progression was determined according to the instructions on the Cell Cycle Analysis kit (BestBio, China). Each experiment was repeated at least thrice.

### Dual-luciferase reporter assay

The 3′-untranslated region (UTR) sequences of circEMB and EGFR and the corresponding mutants were cloned into pmiR-RB-REPORT™ vectors, which were named circEMB-WT, circEMB-MUT, EGFR-WT and EGFR-WUT. The luciferase plasmid was co-transfected with miR-3184-5p mimics or miR-NC. The relative luciferase activity was determined according to the instructions on the Dual-Luciferase Reporter Assay System (Promega, USA).

### RNA immunoprecipitation

RNA immunoprecipitation (RIP) was performed using the Magna RIP Kit (Millipore, Billerica, MA, USA) according to the manufacturer’s instructions. Briefly, OSA cells were lysed in RNA immunoprecipitation (RIP) buffer supplemented with protease inhibitors and RNase. Magnetic beads were pre-incubated with anti-IgG and anti-Ago2 antibodies and were subsequently incubated with the aforementioned cell lysates overnight at 4 °C. Finally, qRT-PCR and western blotting were performed to determine the presence of binding targets.

### RNA pull-down assay using a biotin-labelled probe

C1 magnetic beads were incubated with a biotinylated circEMB probe or oligonucleotide probe (IGE Biotech Co., Ltd., Guangzhou, China) at 25 °C for 2 h. The solution was co-incubated with a cell lysis solution at 4 °C overnight. The precipitates were extracted and purified using an RNeasy Mini Kit (Qiagen, USA) and assessed via qRT-PCR according to the manufacturer’s instructions.

### Subcellular fractionation

The PARIS kit (Invitrogen, USA) was used to isolate nuclear and cytosolic fractions from cells according to the manufacturer’s instructions.

### Bioinformatic analysis

Multiple databases, including ENCORI (http://starbase.sysu.edu.cn/), TargetScan (http://www.targetscan.org/vert_72/), miRanda (http://www.microrna.org/), RNAhybrid (http://bibiserv.techfak.uni-bielefeld.de/rnahybrid/) and RNA22 (https://cm.jefferson.edu/rna22/), were used to predict the interaction between circEMB and miRNA [21–24]. The interaction between miR-3184-5p and EGFR was predicted using TargetScan, miRanda, RNAhybrid and miRDB (http://mirdb.org/) [25]. Pathway enrichment analysis was performed using Reactome in the KOBAS software (KOBAS, Surrey, UK).

### Western blot (WB) analysis

Total protein was isolated from tissues and cells lysed in radioimmunoprecipitation assay (RIPA) buffer (Fudebio, China) and quantified via bicinchoninic acid (BCA) assay. Subsequently, SDS-PAGE (Fudebio, China) was performed to separate proteins of varying molecular weights. The separated proteins on the gel were transferred to a polyvinylidene difluoride (PVDF) membrane (Invitrogen). The membrane was blocked with 5% skim milk at room temperature for 1.5 h and incubated with primary antibodies overnight at 4 °C. The following day, the membrane was incubated with horseradish peroxidase (HRP)-conjugated secondary antibodies (1:10000) (Proteintech, China) at room temperature for 1 h, and protein bands were visualised via enhanced chemiluminescence (Millipore, USA).

### Haematoxylin and eosin (HE) and immunohistochemical (IHC) staining

The extracted fresh animal tissues were washed with normal saline to remove excess blood and immediately fixed in 4% paraformaldehyde overnight. The tissues were embedded in paraffin using an automatic dehydrator and a paraffin-embedding machine and stained with haematoxylin and eosin (H&E) to visualise the histopathological features. Immunohistochemical (IHC) analysis was performed as described previously [20].

### Construction of a xenograft-bearing nude mouse model

All animal experiments were approved by the Ethics Committee of Zhujiang Hospital, Southern Medical University. Nude mice were purchased from the Experimental Animal Center of Southern Medical University. The effects of circEMB on the proliferation of OSA cells were examined using tumour xenograft-bearing mouse models. Stable lentivirus-infected cell lines were isolated, resuspended in 100 μL of PBS (143B, 1 × 106) and injected into the right hindlimb of female nude mice (age, 4 weeks). Malignant growth was examined using an in vivo imaging system. After 4 weeks, mice were euthanised via carbon dioxide inhalation, and tumour tissues were harvested.

To determine the effects of circEMB and its signalling axis on the metastatic capability of OSA cells, 2 × 10^6^ cells (200 uL; 143B) was injected into the tail vein of male nude mice (age, 4 weeks). After 4 weeks, tumour growth in the lung was detected using an in vivo imaging system, and mice were sacrificed via cervical dislocation. Thereafter, lung tissues were harvested for HE staining.

### Molecular docking

AutodockTools (version 1.5.6) was used for molecular docking. The 2D structure of MTX (the ligand) was downloaded from PubMed and converted into a 3D structure using Chem3D. The crystal structure of EGFR was downloaded from Protein Data Bank (PDB) (PDB code: 5UG9) and was used as the protein target for docking simulation. Docking results with the highest score were visualised and analysed using PyMOL.

### Statistical analysis

The SPSS Statistics (version 22.0) software (Chicago, Illinois, USA) was used for statistical analysis. The Mann–Whitney U test or two-tailed Student’s t-test was used to determine the significance of between-group differences, whereas ANOVA followed by Tukey’s test was used to determine the significance of differences among multiple groups. Survival curves were approximated using the Kaplan–Meier approach, and survival data were compared using the log-rank test. Pearson correlation analysis was performed to determine the link between gene expression and clinicopathological features. A *P*-value of < 0.05 was considered significant. Data are expressed as the mean ± standard deviation (SD).

## Results

### Characteristics of circEMB in OSA tissues

**Fig. 1:**
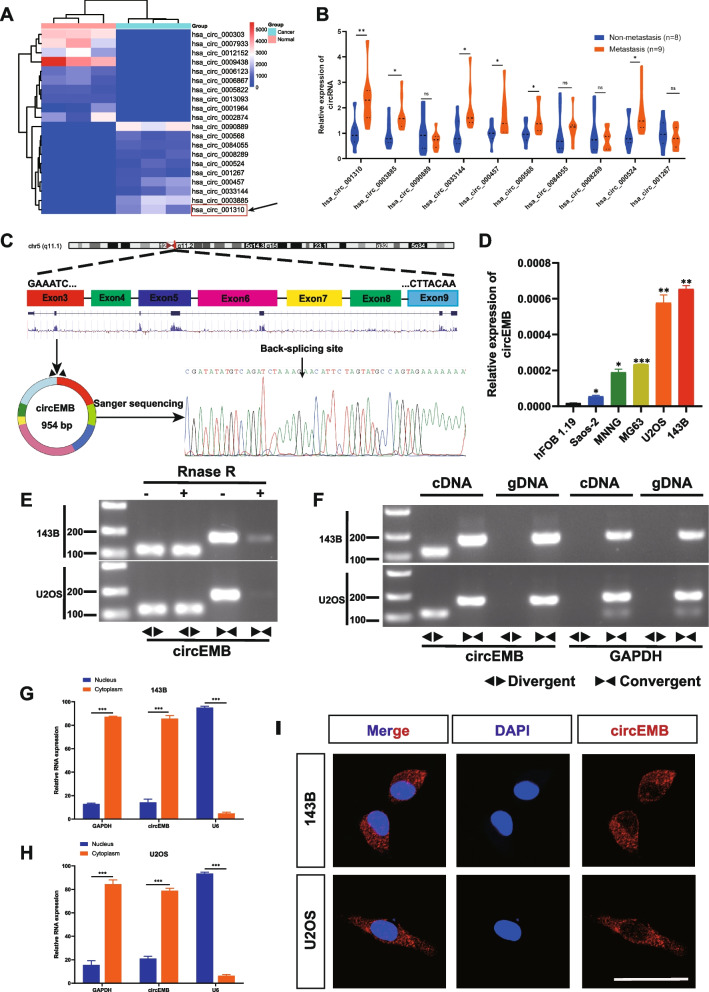
Identification and characterisation of circEMB in OSA cells. **a** Cluster heatmap demonstrating the expression levels of circRNA based on sequencing results. **b** qRT-PCR analysis of candidate genes correlated with tumour invasion and metastasis. **c** Sanger sequencing showing the circEMB loop formation characteristics. The circEMB sequence in the circBASE database was verified to contain seven exons (954) from the EMB gene. **d** Relative expression levels of circEMB in OSA cell lines and normal osteoblasts were determined via qRT-PCR. **e** After treatment with or without RNase R in 143B and U2OS cells, resistance exonuclease digestion of circEMB and EMB were determined by qRT-PCR. **f** The presence of circEMB in 143B and U2OS cells was determined via agarose gel electrophoresis. circEMB was amplified in cDNA instead of gDNA. GAPDH was used as the negative control. **g-h** The expression of circEMB and reference genes (GAPDH and U6) in the cytoplasm and nucleus was verified via nuclear plasma separation assay. circEMB was highly abundant in the cytoplasm. **i** circEMB localisation was analysed via FISH using a Cy3-labelled oligonucleotide. Scale bar, 50 μm. Data are expressed as mean ± SD (*n* = 3) (*, *p* < 0.05; **, *p* < 0.01; ***, *p* < 0.001)

RNA-seq was used to determine circRNA expression in paired OSA tissues and adjacent normal tissues from 3 patients with OSA (Fig. 1a). A total of 404 differentially expressed circRNAs (LogFC > 1, *P* < 0.05) were identified between tumour and normal tissues; of which, 320 were included in the circBase database. Of these 320 circRNAs, 157 were upregulated and 163 were downregulated in tumour tissues. The migration and metastasis of OSA is greatly important indicators for better prognosis, which deserves further studies. To identify circRNAs associated with metastasis, qRT-PCR was performed to determine the expression patterns of the top 10 upregulated circRNAs in the metastatic and non-metastatic tissues of patients (Fig. 1b). The expression of hsa_circ_001310 was found to be significantly different between metastatic and non-metastatic tissues. Based on the results of qRT-PCR and sequencing, we hypothesised that hsa_circ_001310 is an important driver of carcinogenesis. hsa_circ_001310 (referred to as circEMB throughout the article) contains 954 nucleotides and is derived from the EMB gene. Sanger sequencing revealed that circEMB is formed via head-to-tail splicing of exons 3–9 (chromosome 5: 49694940–49,707,217) (Fig. 1c).

The expression of circEMB was found to be high in OSA cells (143B, U2OS, MNNG, MG63 and Saos-2) and hFOB1.19 cells, with the expression being highest in 143B and U2OS cells (Fig. 1d).To investigate the characteristics of the newly discovered circEMB, RNA extract was treated with RNase R, and the stability of circRNA was compared with linear RNA. The results demonstrated that the linear form of EMB was easily degraded by RNase R, whereas the circular form could resist RNase R (Fig. 1e). To rule out both trans-clipping and genome rearrangement, PCR was performed for cDNA and genomic DNA (gDNA) using divergent and convergent primers, respectively, and the findings. Conventionally, the divergent primers could amplify circEMB but not gDNA (Fig. 1f). FISH and cytoplasmic separation experiments revealed that circEMB was predominantly cytoplasmic (Fig. 1g-i). Altogether, these results indicate that circEMB was stable and upregulated in OSA cells.

### circEMB expression in OSA tissues and its relationship with clinical characteristics

**Fig. 2:**
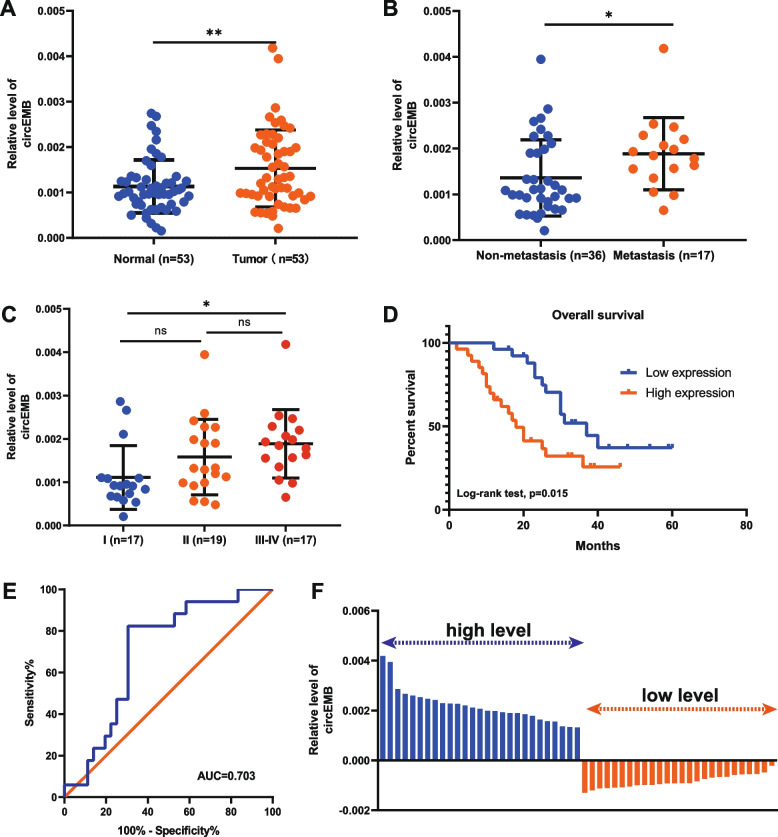
Correlation between circEMB expression and clinical characteristics of patients with OSA. **a** Differential expression of circEMB in OSA tissues and matched adjacent normal tissues. **b** Correlation of circEMB with OSA metastasis. **c** Distribution of circEMB expression in the TNM stage of OSA. **d** Kaplan–Meier analysis indicated that high circEMB expression was correlated with a worse prognosis. **e** ROC curve showing the capability of circEMB in distinguishing patients with metastasis. **f** Based on the median circEMB expression, patients with OSA (*n* = 53) were divided into two groups (high- and low-circEMB-expression groups). Data are expressed as mean ± SD (*n* = 3) (*, *p* < 0.05; **, *p* < 0.01; ***, *p* < 0.001)

The relationship between circEMB expression (53 OSA tissues and 53 matched adjacent normal tissues) and the clinicopathological characteristics of patients was examined. Patients with OSA exhibited significantly upregulated circEMB expression (*P* = 0.00608) (Fig. 2a) (Table 1). Additionally, circEMB expression was correlated with metastasis and clinical stages of OSA (Fig. 2b-c), indicating that the degree of malignancy is correlated with circEMB expression. Receiver operating characteristic (ROC) curve analysis also revealed that circEMB could serve as a biomarker of osteosarcoma metastasis, with an AUC of 0.703 (Fig. 2e). According to the results of qRT-PCR, the samples were categorised into two groups based on the median circEMB expression as follows: low-circEMB-expression (*n* = 26) and high-circEMB-expression (*n* = 27) (Fig. 2f). Kaplan–Meier analysis revealed that survival was worse in the high-circEMB-expression group (Fig. 2d). Altogether, these results suggest that high circEMB expression is associated with the poor prognosis of OSA.

**Table 1:** Correlation between circEMB expression and clinicopathological characteristics of OSA

Parameters	Group	Cases	CircEMB expression	*P* value
Age (years)				0.205
	≤ 18	22	0.001351 ± 0.000720	
	> 18	31	0.001653 ± 0.000920	
Gender				0.274
	Male	32	0.001423 ± 0.000664	
	Female	23	0.001686 ± 0.001070	
Clinical stage				0.018
	I	17	0.001109 ± 0.000735	
	II	19	0.001581 ± 0.000871	
	III-IV	17	0.001886 ± 0.000789	
Distant metastasis				0.005
	Absent	36	0.001358 ± 0.000833	
	Present	17	0.001886 ± 0.000789	
Primary tumor location				0.917
	Arm/hand	19	0.001450 ± 0.000901	
	Leg/foot	29	0.001551 ± 0.000846	
	Others	5	0.001684 ± 0.000795	

### circEMB promotes the proliferation, migration and invasion capabilities of OSA cells in vitro

**Fig. 3:**
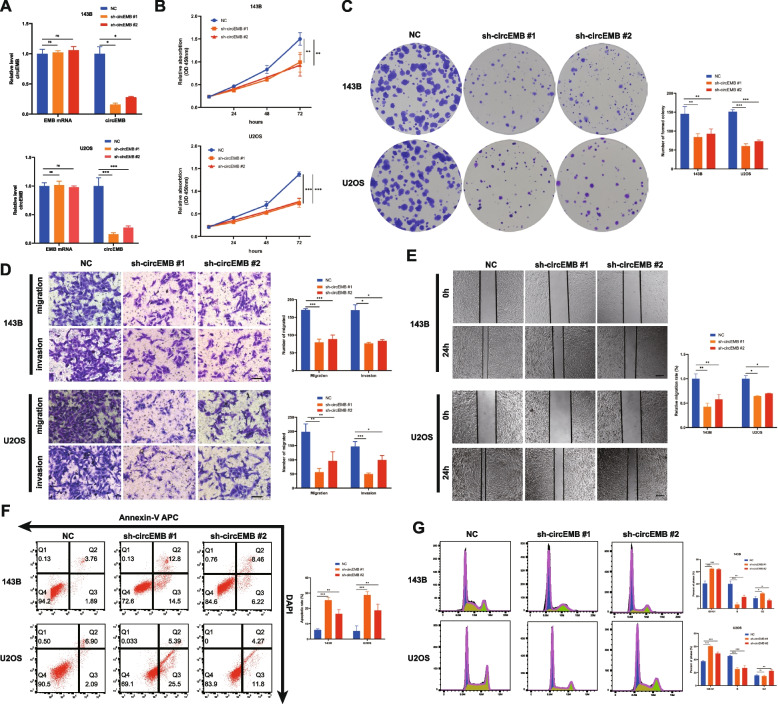
CircEMB promotes OSA cell proliferation, migration, invasion, apoptosis and cell cycle distribution. **a** Two shRNAs were designed to silence circEMB expression without affecting the mRNA expression of EMB. sh-circEMB #1 had the highest silencing efficiency. **b** CCK-8 assay revealed that circEMB knockdown inhibited the proliferation of 143B and U2OS cells. **c** Colony formation assay revealed that circEMB knockdown inhibited the proliferation of 143B and U2OS cells. **d** Transwell assay was performed to assess the influence of circEMB knockdown on the invasion and migration capabilities of 143B and U2OS cells. Scale bar, 100 μm. **e** Wound-healing assay was used to assess the effects of circEMB knockdown on 143B and U2OS cell migration. Scale bar, 200 μm. **f** Effects of circEMB silencing on apoptosis were examined via APC annexin V/PI staining as well as flow cytometry. **g** Flow cytometry was performed to assess the effects of circEMB on the cell cycle of 143B and U2OS cells. Data are expressed as mean ± SD (*n* = 3) (*, *p* < 0.05; **, *p* < 0.01; ***, *p* < 0.001)

Several in vitro experiments were performed to examine the regulatory effects of circEMB on OSA cells. Two shRNAs targeting circEMB were constructed, and their knockout efficiency was verified in the two cell lines (143B and U2OS) with the highest expression of circEMB, without affecting the mRNA expression of EMB (Fig. 3a). The results showed that shRNA-1 not only had the highest knockout efficiency in 143B and U2OS cells but also had a significantly greater effect on OSA cells than shRNA-2. CCK-8 (Fig. 3b) and clone formation (Fig. 3c) assays revealed that the proliferation of OSA cells was inhibited after circEMB knockdown. Transwell and wound healing assays revealed that circEMB knockdown remarkably suppressed the migration and invasion abilities of OSA cells (Fig. 3d-e). Additionally, flow cytometry showed that circEMB knockdown induced apoptosis in OSA cells (Fig. 3f) and resulted in G1/S-phase arrest (Fig. 3g). shRNA-1 was selected for further analysis because its inhibitory effects on the malignant behaviour of OSA were more prominent. Altogether, these results suggest that circEMB promotes the malignant behaviour of OSA.

### circEMB serves as an miR-3184-5p sponge

**Fig. 4:**
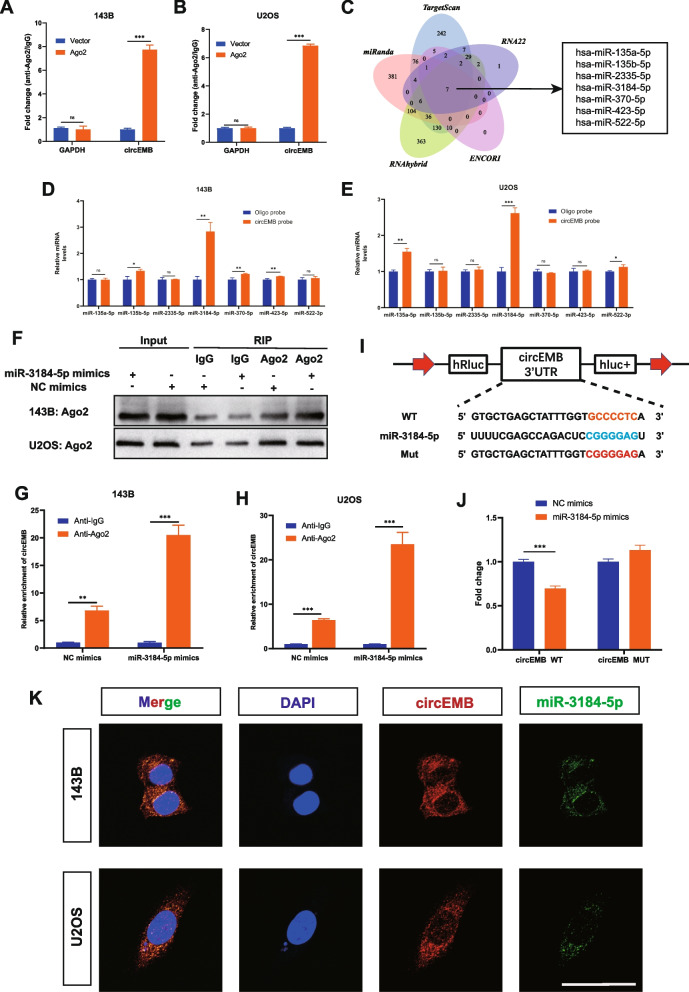
CircEMB sponges miR-3184-5p. **a-b**RIP assay was performed to verify the binding between circEMB and the Ago2 protein. **c** Schematic representation of the overlap among target miRNAs of circEMB predicted using the TargetScan, miRanda, RNAhybrid, ENCORI and RNA22 databases. **d-e** RNA pull-down assay verified the binding between seven candidate miRNAs and circEMB. **f-h** RIP assay of miRNA showed that in contrast to the NC group, the miR-3184-5p mimics group effectively suppressed circEMB levels in 143B and U2OS cells. **i-j** HEK293T cells were co-transfected with miR-3184-5p mimics and wild-type or mutant circEMB luciferase reporter vector, and luciferase reporter activity was assessed. **k** Subcellular colocalization between circEMB (red) and miR-3184-5p (green) was observed in in 143B and U2OS by confocal FISH. Scale bar, 50 μm. Data are expressed as mean ± SD (*n* = 3) (*, *p* < 0.05; **, *p* < 0.01; ***, *p* < 0.001)

Given that circEMB localises in the cytoplasm, it may function as a molecular sponge of miRNAs after transcription. Therefore, RIP assay was performed using Ago2 to examine the role of circEMB in sponging miRNAs. The results revealed that circEMB expression was remarkably increased in the Ago2 group, indicating that circEMB binds to miRNAs via the Ago2 protein (Fig. 4a-b).

Two online databases, namely, RNA22 and ENCORI, and three local databases, namely, TargetScan, miRanda and RNAhybrid, were used to predict the miRNA targets of circEMB, and seven miRNAs (hsa-miR-135a-5p, hsa-miR-135b-5p, hsa-miR-2335-5p, hsa-miR-3184-5p, hsa-miR-370-5p, hsa-miR-423-5p, hsa-miR-522-5p) were eventually identified (Fig. 4c). However, the binding mechanism of the same circRNA to the presence of different miRNAs in different tumors affects cell growth [26–28]. Therefore, we need to explore the specific miRNA that circEMB binds in OSA. Next, we used biotinylated circEMB probes to determine the mode of interaction between circEMB and the seven predicted miRNAs to identify more stable interactions between them in OSA. Finally, we found that circEMB and miR-3184-5p may have a stronger and more stable relationship (Fig. 4d-e).

Given that miR-3184-5p plays a substantial role in tumorigenesis in OSA, we examined whether it can act as an Ago2 sponge. To ensure the validity of subsequent miR-3184-5p-related experiments, we validating the transfection efficiencies of the miR-3184-5p mimic and inhibitor (Additional file 2: Fig. S1A-B). RIP assays with anti-Ago2 and anti-IgG revealed that miR-3184-5p overexpression increased the adsorption of circEMB on anti-Ago2 instead of anti-IgG (Fig. 4f-h). The dual-luciferase reporter assay further verified the binding between circEMB and miR-3184-5p (Fig. 4i-j). Meanwhile, RNA-FISH experiment results showed that circEMB and miR-3184-5p were co-localized in the cytoplasm of OSA (Fig. 4k).

Altogether, these results indicate that circEMB forms a complex with Ago2 and affects Ago2-mediated miR-3184-5p-guided RNAi and Ago2-dependent miRNA biogenesis.

### Inhibition of miR-3184-5p mediates the oncogenic function of circEMB

Owing to the lack of studies on miR-3184-5p in OSA, we validated its expression profile in clinical OSA samples (*n* = 53) via qPCR and examined whether miR-3184-5p expression was associated with the clinicopathological features of patients with OSA. The results revealed that the loss of miR-3184-5p was associated with advanced pathological stages, TNM stage and invasion depth (*P* < 0.05) (Table 2) (Additional file 3: Fig. S2A-B). We categorized the enrolled patients into the high-expression group (*n* = 27) and low-expression group (*n* = 26) according to the median value of miR-3184-5p expression in OSA tissues (Additional file 3: Fig. S2C). Kaplan-Meier survival analysis (log-rank test) exhibited that high expression levels of miR-3184-5p indicate good prognostic outcomes (Additional file 3: Fig. S2D). Additionally, a negative correlation was observed between the expression of miR-3184-5p and circEMB (Additional file 3: Fig. S2E). Consistently, miR-3184-5p expression was lower in OSA cell lines (143B, U2OS, MNNG, MG63 and Saos-2) than in the normal human osteoblast cell line hFOB1.19 (Additional file 3: Fig. S2F). These results indicate that miR-3184-5p plays an important role as a tumour suppressor.

To explore the functional role of miR-3184-5p, we transfected miR-3184-5p mimics into 143B and U2OS cells. All the results displayed that miR-3184-5p could significantly affect the ability of proliferation, migration, invasion, apoptosis and cell cycle arrest of OSA cells (Additional file 4: Fig. S3A-K). These results further demonstrate that high expression of miR-3184-5p suppresses OSA progression and metastasis.

**Fig. 5:**
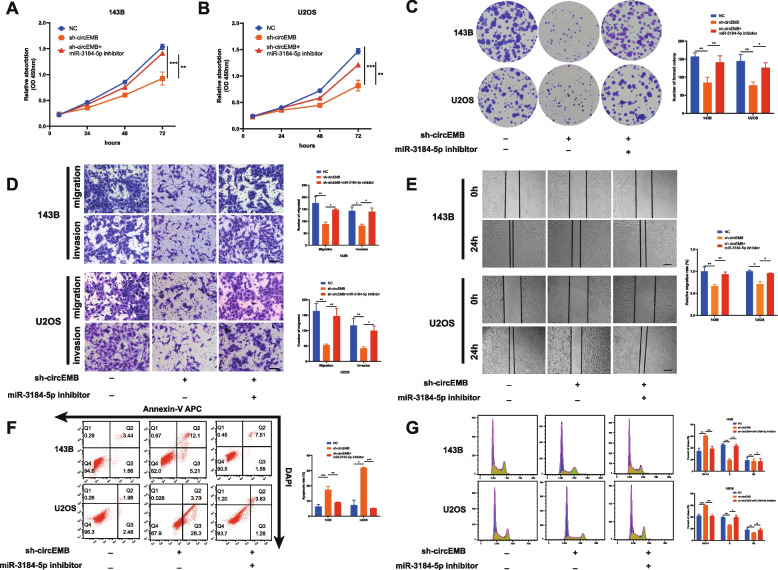
CircEMB affects the malignant behaviors of OSA by sponging miR-3184-5p. **a-c** CCK-8 and colony formation assays showed that miR-3184-5p knockdown partly reversed circEMB knockdown-induced decrease in cell proliferation. **d-e** Transwell and wound healing assays revealed the mechanisms through which miR-3184-5p knockdown partly reversed circEMB-mediated invasion and migration abilities of 143B and U2OS cells. Scale bar, 100 μm Scale (Transwell); Scale bar, 200 μm(wound-healing assays). **f** Flow cytometry revealed that silencing miR-3184-5p partly reversed circEMB-mediated apoptosis of 143B and U2OS cells. **g** Flow cytometry revealed that silencing miR-3184-5p partly reversed circEMB-mediated G1/S phase arrest of 143B and U2OS cells. Data are expressed as mean ± SD (*n* = 3) (*, *p* < 0.05; **, *p* < 0.01; ***, *p* < 0.001)

Based on the abovementioned results, we hypothesised that circEMB affects the occurrence of OSA via miR-3184-5p. To test this hypothesis, we treated circEMB-knockdown cells with an inhibitor of miR-3184-5p and subsequently performed CCK-8 and colony formation assays (Fig. 5a-c). The miR-3184-5p inhibition was found to reverse the circEMB knockdown-induced decrease in the proliferation of 143B and U2OS cells. Transwell and wound healing assays revealed that miR-3184-5p inhibition attenuated the circEMB knockdown-induced decrease in the migration and invasion abilities of 143B and U2OS cells (Fig. 5d-e). Compared with circEMB knockdown alone, combined circEMB knockdown and miR-3184-5p inhibition significantly inhibited apoptosis and cell cycle arrest (Fig. 5f-g). Altogether, these results suggest that circEMB promoted tumour progression in OSA primarily by inhibiting the antitumour effects of miR-3184-5p.

### circEMB regulates EGFR expression via miR-3184-5p

**Fig. 6:**
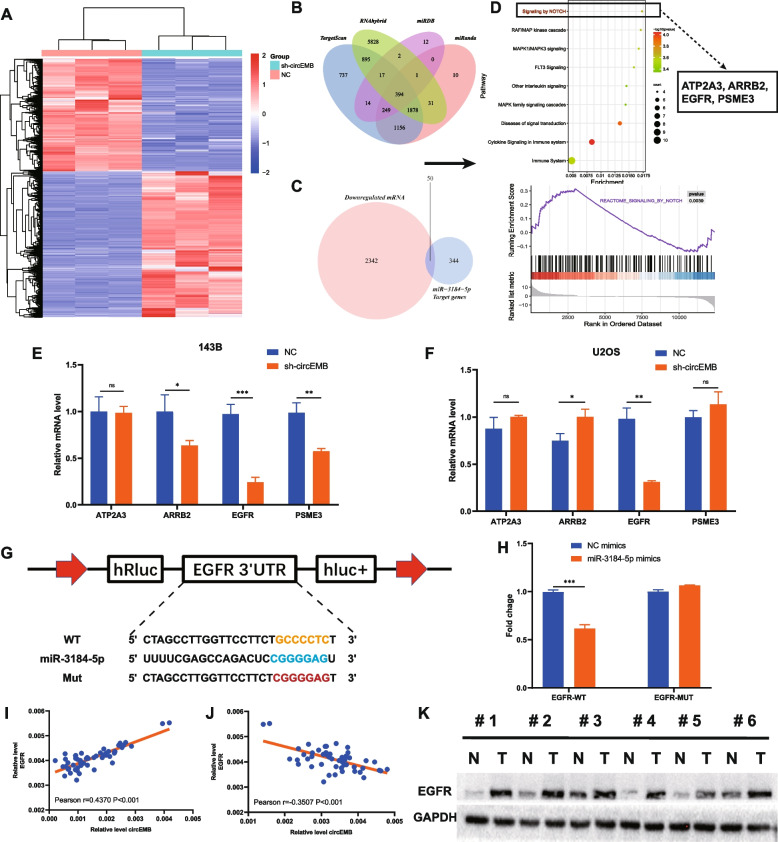
The circEMB/miR-3184-5p/EGFR axis was activated in OSA cells. **a** 143B cells transfected with sh-circEMB and scramble shRNA were subjected to RNA-seq. **b** Schematic representation of the overlap among target mRNAs of miR-3184-5p predicted using the TargetScan, miRanda, RNAhybrid, and miRDB databases. **c** Schematic representation of the overlap among target mRNAs of miR-3184-5p and mRNAs downregulated via circEMB knockdown. **d** Pathway enrichment analysis and GSEA revealed that the overlap genes were markedly enriched in the Notch signalling pathway. **e-f** After transfection of sh-circEMB into 143B and U2OS cells, the relative expression of four candidate mRNAs was determined via qRT-PCR. **g-h** Verification of the binding between miR-3184-5p and EGFR in HEK293T cells by dual-luciferase reporter gene assay. **i-j** Correlation between the mRNA expression of circEMB and EGFR and between that of miR-3184-5p and EGFR in 53 OSA samples. **k** Expression of EGFR in paired OSA and adjacent normal tissues was examined via western blotting. Data are expressed as mean ± SD (*n* = 3) (*, *p* < 0.05; **, *p* < 0.01; ***, *p* < 0.001)

circRNAs sponge miRNAs to prevent them from exerting regulatory effects on target genes. RNA sequencing was performed after silencing circEMB to identify the downstream target genes regulated by circEMB (Fig. 6a). Because circRNAs function as miRNA sponges to attenuate the miRNA-induced suppression of downstream target genes, like cricRNAs, have co-directional expression. Additionally, TargetScan, miRanda, RNAhybrid and miRDB were used to identify the target genes of miR-3184-5p. The results revealed 394 genes as the direct targets of miR-3184-5p (Fig. 6b); of which, 50 genes were selected for subsequent experiments. These 50 genes were used for pathway enrichment analysis using KOBAS to identify significant signalling pathways involved in the regulation of the circEMB/miR-3184-5p axis (Fig. 6c). Pathway enrichment analysis and GSEA revealed that the target genes were primarily associated with the Notch signalling pathway (Fig. 6d). Thereafter, four genes involved in the Notch signalling pathway, namely, EGFR, ARRB2, PSME3 and ATP2A3, were identified as potential regulatory targets of the circEMB/miR-3184-5p axis. The expression patterns of the target genes in circEMB-knockdown OSA cells (143B and U2OS) revealed that EGFR expression was more closely associated with circEMB expression (Fig. 6e-f). Because miRNAs always target the 3′-UTR of mRNAs, the sequences of miR-3184-5p and the 3′-UTR of EGFR were compared. Dual-luciferase reporter assay revealed a specific binding site for miR-3184-5p in the 3′-UTR of EGFR (Fig. 6g-h). Pearson analysis revealed that EGFR expression was positively correlated with circEMB expression and negatively correlated with miR-3184-5p expression in OSA tissues (Fig. 6i-j). Additionally, EGFR expression was significantly higher in OSA tissues than in paracancerous tissues (Fig. 6k). Altogether, these results suggest that EGFR is a direct target of miR-3184-5p.

### circEMB promotes OSA progression and chemoresistance via the circEMB/miR-3184-5p/EGFR axis

**Fig. 7:**
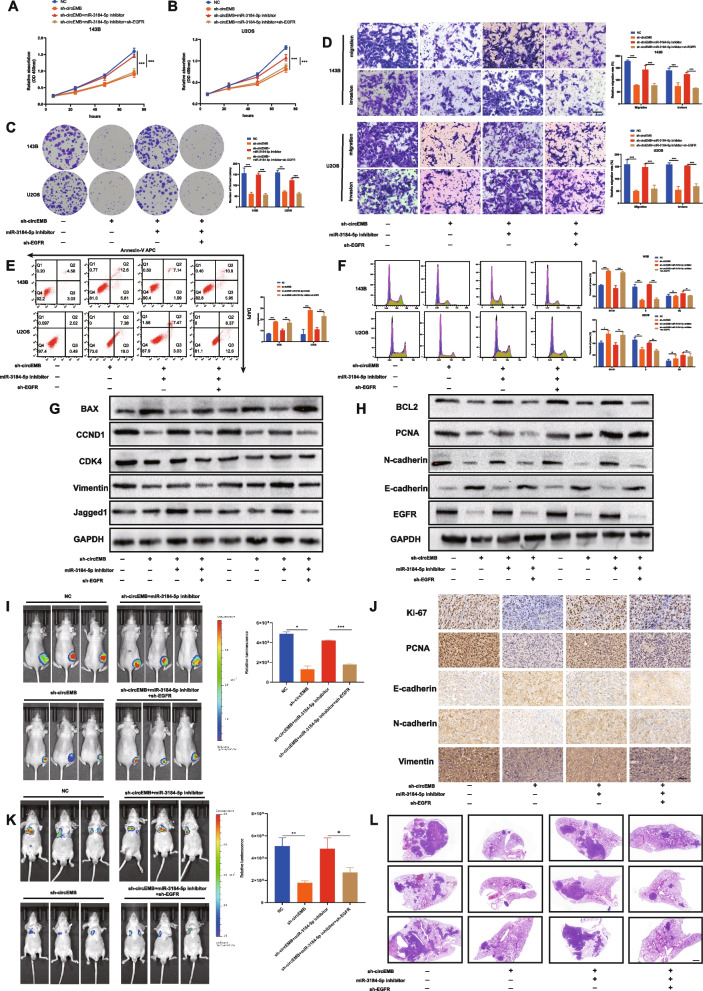
circEMB affects the malignant behaviour of OSA via the circEMB/miR-3184-5p/EGFR axis. **a-c** CCK-8 and colony formation assays revealed the changes in proliferation abilities of 143B and U2OS after simultaneous cell transfection with sh-circ-EMB, miR-3184-5p inhibitors, and sh-EGFR. **d** Transwell assay was performed to examine the effects of the circEMB/miR-3184-5p/EGFR axis on the migration and invasion abilities of cells. Scale bar, 100 μm. **e-f** Effects of the circEMB/miR-3184-5p/EGFR axis on cell cycle arrest and apoptosis were evaluated by flow cytometry. **h** Western blotting was used to assess the effects of the circEMB/miR-3184-5p/EGFR axis on the expression of E-cadherin, N-cadherin, Vimentin, PCNA, CDK4, CCND1, BAX, BCL2, Jagged1, and EGFR. **i** An in vivo imaging system was used to examine the effects of the circEMB/miR-3184-5p/EGFR axis on cell proliferation. **j** IHC staining for Ki-67, PCNA, Vimentin, N-cadherin and E-cadherin in each group. Scale bar, 100 μm. **k** An in vivo imaging system was used to examine the effects of the circEMB/miR-3184-5p/EGFR axis on metastasis in nude mice. Scale bar, 1000 μm. **l** Microscopic images of HE staining of metastatic nodules in the lungs of nude mice. Data are expressed as mean ± SD (*n* = 3) (*, *p* < 0.05; **, *p* < 0.01; ***, *p* < 0.001)

We hypothesised that circEMB can antagonise miR-3184-5p. To verify this hypothesis, we examined whether circEMB affects EGFR expression via miR-3184-5p, thus regulating the occurrence and progression of OSA. Rescue experiments were performed via circEMB knockdown, EGFR knockdown and miR-3184-5p inhibition to examine whether circEMB acts as an oncogene by modulating EGFR. CCK-8 assay, transwell migration and invasion assay and flow cytometry revealed that circEMB downregulation reversed the effects of miR-3184-5p inhibition on the proliferation, migration and invasion abilities; apoptosis and cell cycle arrest of OSA cells, whereas EGFR knockdown reversed the circEMB and miR-3184-5p knockdown-induced cell functional phenotype (Fig. 7a-f). Additionally, the results of western blotting validated that the circEMB/miR-3184-5p/EGFR axis regulated the expression of cell proliferation-related proteins (PCNA), apoptosis-related proteins (BAX and BCL2), cell cycle-related proteins (CDK4 and CCND1) and epithelial–mesenchymal transformation-related proteins (Vimentin, E-cadherin and N-cadherin) by affecting EGFR expression. Western blotting also revealed that the circEMB/miR-3184-5p/EGFR axis has the potential to influence the Notch signalling pathway (EGFR and Jagged1) (Fig. 7g-h). In vivo experiments showed that the circEMB/miR-3184-5p/EGFR axis affected OSA of capable for subcutaneous tumour formation (Fig. 7i). IHC analysis revealed that circEMB knockdown decreased the protein expression of Ki-67, PCNA, N-cadherin and Vimentin but increased the protein expression of E-cadherin. However, silencing the miR-3184-5p/EGFR axis partially reversed these effects (Fig. 7j). In vivo imaging of the tail vein (in mouse models of lung metastasis) showed that the circEMB/miR-3184-5p/EGFR axis affected OSA of capable for metastasis (Fig. 7k). HE staining further validated these results (Fig. 7l). Collectively, Altogether, these results suggest that circEMB promotes the malignant behaviour of OSA by regulating the miR-3184-5p/EGFR axis.

The Notch pathway is tightly associated with the chemosensitivity of tumour cells. Because the circEMB/miR-3184-5p/EGFR axis affects the development of OSA via the Notch signalling pathway, we examined the effects of circEMB on the chemoresistance of OSA and verified the results using MTX.

**Fig. 8:**
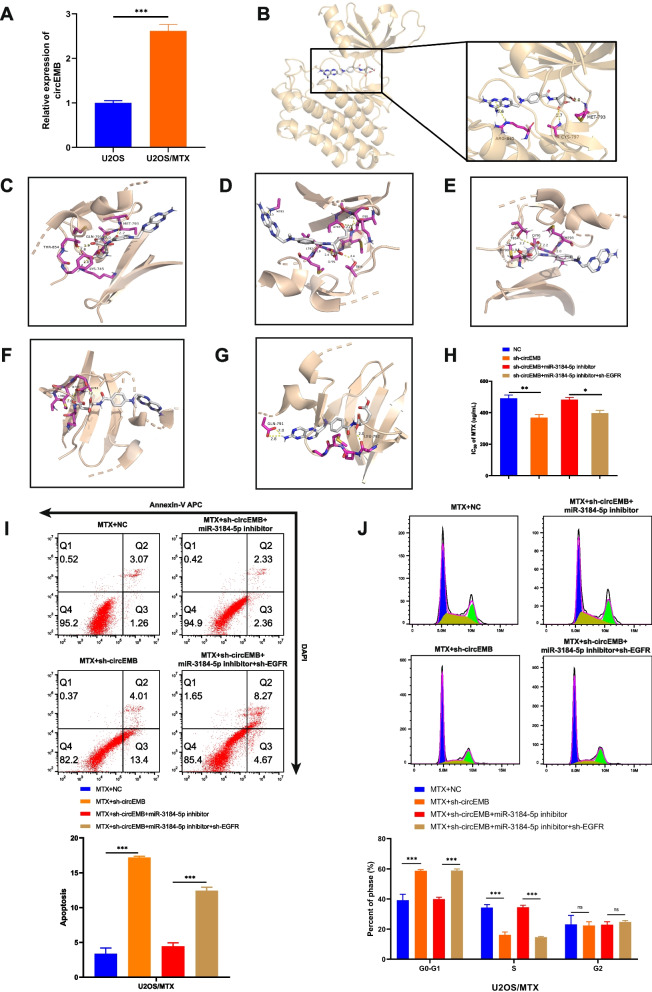
The circEMB/miR-3184-5p/EGFR axis regulates the sensitivity of OSA cells to MTX. **a** circEMB expression was upregulated in MTX-resistant U2OS cells. **b-g** AutoDock Vina was used for the molecular docking of MTX and EGFR. **h** The IC50 of OSA cells treated with MTX. **i-k** Flow cytometric analysis of apoptosis and cell cycle distribution of MTX-resistant U2OS cells treated with MTX. Data are expressed as mean ± SD (*n* = 3) (*, *p* < 0.05; **, *p* < 0.01; ***, *p* < 0.001)

qRT-PCR revealed that circEMB expression was higher in the MTX-resistant OSA cell line U2OS (U2OS/MTX cells) (Fig. 8a). A study from Austria showed that gefitinib, an EGFR inhibitor, in combination with MTX can increase the sensitivity of OSA cells to MTX [29]. Therefore, we examined the relationship between EGFR and MTX. Molecular docking showed that EGFR and MTX had robust interactions and strong binding affinity (− 7.9 kcal/mol). The amino acid residues ALA871, LYS913, LEU718 and GLY796 on the binding domain of EGFR were found to bind to MTX via hydrophobic interactions. The amino acid residues ALA743, LEU844, VAL726, LEU792, GLN791, PHE856, ASN842, LYS875, ARG858, PRO877, GLY721, GLY719 and GLY796LEU718 of EGFR were found to bind to MTX via van der Waals forces. Additionally, MTX formed π-π interactions with the amino acid residue PHE723 of EGFR (Fig. 8b-g). To explore the potential role of circEMB/miR-3184-5p/EGFR axis on MTX resistance in OSA, we perturbed the expression of circEMB/miR-3184-5p/EGFR axis in U2OS/MTX. We found that the IC50 of U2OS/MTX to MTX decreased after circEMB expression was reduced, and the resistance phenotype of U2OS/MTX was reversed after miR-3184-5p knockdown. Similarly, we found that EGFR as part of the biological axis also had an effect on the IC50 of MTX (Fig. 8h). Flow cytometry revealed that the circEMB/miR-3184-5p/EGFR axis regulated drug sensitivity via apoptosis and cell cycle regulation (Fig. 8i-j).

## Discussion

At present, the management strategies for newly diagnosed OSA include individualised high-dose neo-adjuvant chemotherapy (also called preoperative chemotherapy), surgery and adjuvant chemotherapy after surgery. However, owing to metastasis and drug resistance, the 5-year survival rate of patients with OSA is as low as 20% [30]. Therefore, it is of clinical significance to elucidate cellular mechanisms underlying the development of OSA and develop new treatment strategies for improving the prognosis.

Epigenetic alterations, including DNA and m6A methylation, and noncoding RNAs involved in tumour development, invasion, metastasis and drug resistance play an important role in guiding clinical treatment [31–35]. circRNAs, noncoding RNAs, are potential biomarkers for evaluating tumour metastasis and drug resistance outcomes in several cancers, including hepatocellular carcinoma, breast cancer and glioma [36–39]. However, the mechanisms through which circRNAs affect the development, metastasis and drug resistance of OSA remain unclear.

**Fig. 9:**
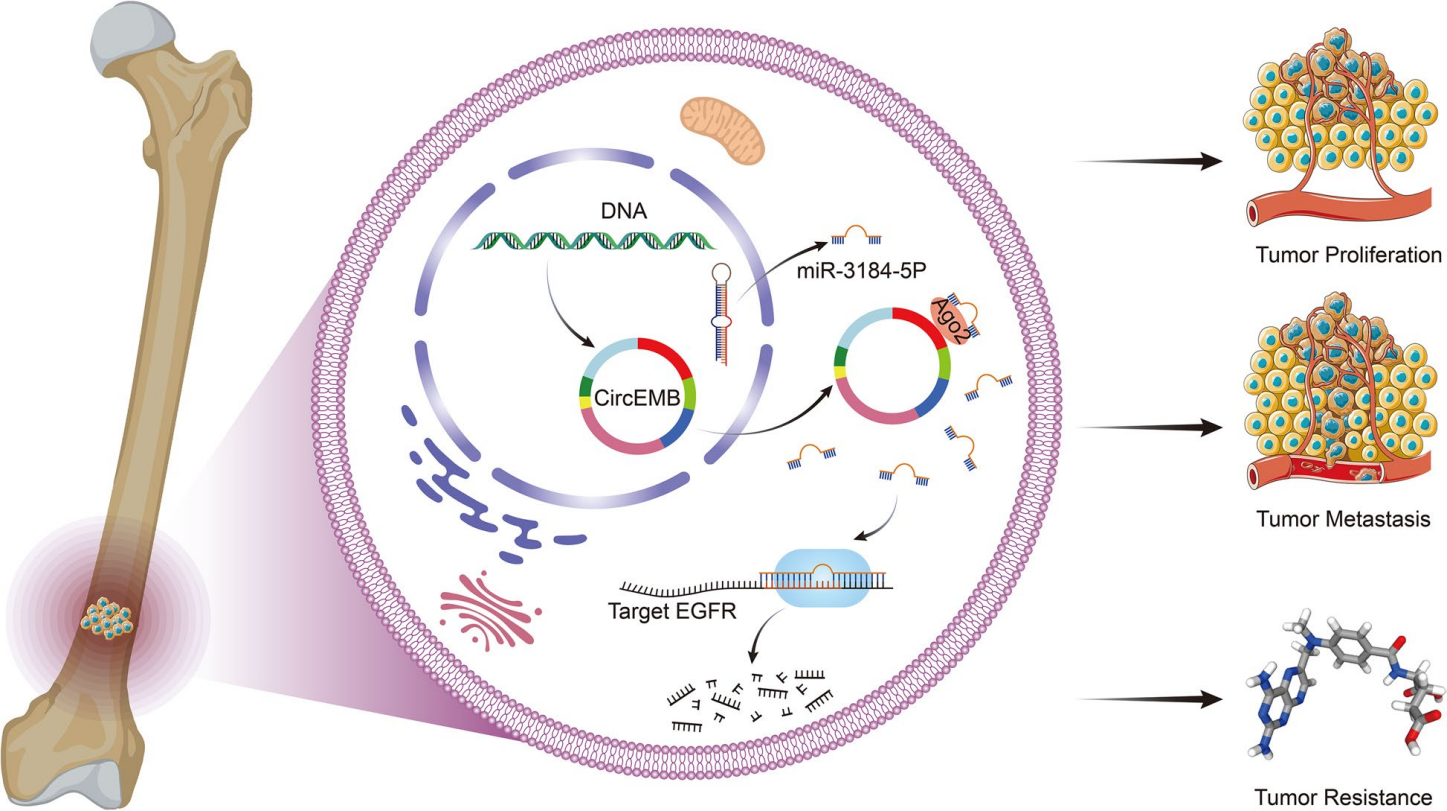
Schematic representation of the effects of the circEMB/miR-3184-5p/EGFR axis on the malignant behaviour of OSA and its resistance to MTX

This study revealed that the circEMB/miR-3184-5p/EGFR axis regulates the progression of OSA by regulating cell proliferation, tumour invasion and metastasis, apoptosis and cell cycle arrest. Additionally, the circEMB/miR-3184-5p/EGFR axis is involved in resistance to MTX (Fig. 9).

A fundamentally novel circRNA, named circEMB, was identified via high-throughput sequencing of tissue samples collected from patients with OSA. circEMB is upregulated in OSA tissues and promotes the proliferation, migration and invasion abilities of OSA cells. To the best of our knowledge, this study is the first to report that circEMB promotes the malignant behaviour of OSA.

circEMB localises in the cytoplasm and is significantly associated with miRNAs. Downstream miRNAs are important agents through which circRNAs affect the target genes. However, most studies investigating the relationship between circRNAs and miRNAs lack systematic experimental verification and mainly focus on identifying candidate miRNAs via bioinformatic analysis [40]. In this study, miR-3184-5p was identified as the target miRNA of circEMB. Importantly, circEMB was verified to bind the 3’UTR region of miR-3184-5p through Ago2.

miR-3184-5p inhibits the malignant behaviour of various tumours [41–44]. However, previous studies have not reported the role of miR-3184-5p in tumorigenesis in OSA. To the best of our knowledge, this is the first study to report that miR-3184-5p is found in OSA tissues and cells and is negatively associated with circEMB expression. circEMB can sponge miR-3184-5p to rescue the functions of downstream genes that affect the malignant behaviour of OSA.

Furthermore, EGFR was identified as a potential functional target of miR-3184-5p. EGFR, an epidermal growth factor family member, is closely associated with cell proliferation, apoptosis and tumour invasion and metastasis in OSA [45–48], which is consistent with the findings of this study. Increased EGFR expression was positively correlated with circEMB expression and negatively correlated with miR-3184-5p expression in OSA tissues and cells. Functionally, after successive circEMB and miR-3184-5p knockdown, the promotion of cell proliferation and invasion triggered by EGFR knockdown was remarkably reversed. These results suggest that circEMB affects EGFR expressions by sponging miR-3184-5p, thereby accelerating OSA progression.

Clinically, MTX is a front-line chemotherapeutic agent for patients with OSA [49]. However, many patients with OSA exhibit primary and/or acquired resistance to MTX. In this study, circEMB was found to be highly expressed in MTX-resistant cells. Inhibiting the function of EGFR can alter the resistance of OSA to MTX [29]. In this study, molecular docking revealed an interaction between EGFR and MTX. Based on the findings of this study and those of previous studies, we speculated that suppressed EGFR expression inhibits EGFR and alters the sensitivity of tumour cells to MTX. Experimentally, we found that the circEMB/miR-3184-5p/EGFR axis can affect drug resistance in OSA.

This retrospective study has several limitations, and there is scope for future work. First, the mouse model of OSA was not used in situ; therefore, we could not completely simulate a real tumour microenvironment of OSA in vivo. Second, the molecular mechanisms through which the circEMB/miR-3184-5p/EGFR axis regulates MTX resistance warrant further investigation.

## Conclusions

CircEMB, a previously unrecognised oncogenic driver, is upregulated in OSA tissues and is associated with cell proliferation, migration and invasion in OSA. In addition, we propose the mechanism by which circEMB can block the EGFR inhibitory activity of miR-3184-5p. The circEMB/miR-3184-5p/EGFR axis affects the sensitivity of OSA cells to MTX. Therefore, circEMB is a candidate prognostic biomarker and therapeutic target for OSA.

## Supplementary Information

**Additional file 1:** **Table S1.** Primer sequences for qRT-PCR in this study.

**Additional file 2:** **Fig. S1.** Verification of transfection efficiency of miR-3184-5p in OSA cells.
(A) After the transfection of mimic and inhibitor of miR-3184-5p, qRT-PCR was used to detect the transfection efficiencies. Data are expressed as mean ± SD (*n* = 3) (*, *p* < 0.05; **, *p* < 0.01; ***, *p* < 0.001).

**Additional file 3: Fig. S2.** The relationship between the miR-3184-5p and clinicopathological characteristics. (A) Comparison of miR-3184-5p expression between non-metastatic and metastatic OSA tissues. (B) Distribution characteristics of miR-3184-5p expression in the TNM stage of OSA. (C) In the 53-patients cohort, according to the median value of miR-3184-5p expression, they were divided into high expression group and low expression group. (D) Overall survival analysis of patients with low and high expression of miR-3184-5p using log-rank test and Kaplan–Meier analysis. (E) Correlation analysis between circEMB and miR-3184-5p in OSA tissues. (F) Relative miR-3184-5p levels in OSA cell lines and normal osteoblasts were ascertained by qRT-PCR. Data are expressed as mean ± SD (n = 3) (*, *p* < 0.05; **, *p* < 0.01; ***, *p* < 0.001).

**Additional file 4: Fig. S3.** Overexpression of miR-3184-5p was associated with decreased OSA cell migration and invasion, nor increased apoptosis and G1/S phase arrest. (A-B) The proliferation ability of OSA cells after overexpression of miR-3184-5p was determined by CCK-8. (C-F) Transwell assays detected the changes of invasion and metastasis ability of OSA cells with or without miR-3184-5p overexpression. (C and E) Refer to 143B, while (D and F) refer to U2OS. Scale bar, 100 μm. (G-H) Flow cytometric apoptosis analysis detected the changes of apoptosis of OSA cells with or without miR-3184-5p overexpression. (I-K) Flow cytometry assay showed the regulation of cell cycle by overexpressing miR-3184-5p. Data are expressed as mean ± SD (n = 3) (*, *p* < 0.05; **, *p* < 0.01; ***, *p* < 0.001).

## Data Availability

The datasets used and/or analysed in the present study are available from the corresponding author upon reasonable request.

## Abbreviations

OSA: Osteosarcoma
CircRNAs: Circular RNAs
circEMB: hsa_circ_001310
miR-3184-5p: MicroRNA-3184-5p
MTX: Methotrexate
cDNA: Complementary DNA
gDNA: Genomic DNA
shRNAs: Short hairpin RNAs
FISH: Fluorescence in situ hybridization
Ago2: Argonaute
RIP: RNA immunoprecipitation
HE: Haematoxylin and eosin
IHC: Immunohistochemical
EGFR: Epidermal growth factor receptor
PCNA: Proliferating cell nuclear antigen
BAX: B-cell leukaemia/lymphoma 2-associated X
BCL2: B-cell leukaemia/lymphoma 2
CCND1: Cyclin D1
CDK4: Cyclin-dependent kinase 4
Jagged1: Jagged canonical Notch ligand 1
