# Supplementary material for: Circular RNA circEMB promotes osteosarcoma progression and metastasis by sponging miR-3184-5p and regulating EGFR expression
Source: Biomark Res. 2023 Jan 7;11:3. doi: 10.1186/s40364-022-00442-9 (PMC9825012; doi:10.1186/s40364-022-00442-9)
Supplement: Supplementary file 1 — Additional file 1: Table S1. Primer sequences for qRT-PCR in this study. [file 40364_2022_442_MOESM1_ESM.docx]

**Additional file 1:** **Table S1.** Primer sequences for qRT-PCR in this study.

**Table S1** Primer sequences for qRT-PCR in this study

| Gene | Forward primer (5’ to 3’) | Reverse primer (5’ to 3’) |
| --- | --- | --- |
| CircEMB | CGATATATGTCAGATCTAAAGAACAT | GTTGTGAACTGGCATGTGAGA |
| EMB  hsa_circ_0003885 | ATGGGGAATCTTACTGGTGCC  TGGCAAGGAATTTCAGGCAG | CCCCTCATCTGAGTGCTTCTTT  AGGGCTTGCTCTCCATGATG |
| hsa_circ_0090889 | GGAACAGCTGATCAGTGTGC | CCTGGGCAGTCACCTTCTTA |
| hsa_circ_0033144 | GGAGAACATTGCAGCAGAGG | CACTGCTTCCTTTTGTGCTCT |
| hsa_circ_000457 | CCCCAGCTCGATATGTGTCA | TGGGTCAAAAGTGCTCTGTG |
| hsa_circ_000568 | CTGTTCTGGAGGCTGTGGAA | AAGTCAGCTTTGGTCCTCC |
| hsa_circ_0084055 | TAGCGACAATGTGGACCTGC | AGTCTTTGCAGCACAGTCCT |
| hsa_circ_0008289 | GATGCGACGGATCAGTGACT | CCATGAAGTGAGCCTGGAGT |
| hsa_circ_000524 | CCAATGGAAGTGTTTAAGCAATC | GCTGTCCTCGAACCAGTCAA |
| hsa_circ_001267 | CAGGACTCCAAGGTCATTTTCG | AAGTAGCCATGCAGCTGGG |
| miR-3184-5p | ACACTCCAGCTGGGTGAGGGGCCTCAGACCGA | TGGTGTCGTGGAGTCG |
| ATP2A3 | CTGGTCATCATGCTGATCCTC | CAGCGTGGTGGACTTGATCT |
| ARRB2 | TCCATGCTCCGTCACACTG | ACAGAAGGCTCGAATCTCAAAG |
| EGFR | AGGCACGAGTAACAAGCTCAC | ATGAGGACATAACCAGCCACC |
| PSME3 | AAGGTTGATTCTTTCAGGGAGC | AGTGGATCTGAGTTAGGTCATGG |
| U6 | CTCGCTTCGGCAGCACA | AACGCTTCACGAATTTGCGT |
| GAPDH | CTCCAAAATCAAGTGGGGCG | TGGTTCACACCCATGACGAA |
