# Supplementary figures and images for: Circular RNA circEMB promotes osteosarcoma progression and metastasis by sponging miR-3184-5p and regulating EGFR expression
Source: Biomark Res. 2023 Jan 7;11:3. doi: 10.1186/s40364-022-00442-9 (PMC9825012; doi:10.1186/s40364-022-00442-9)

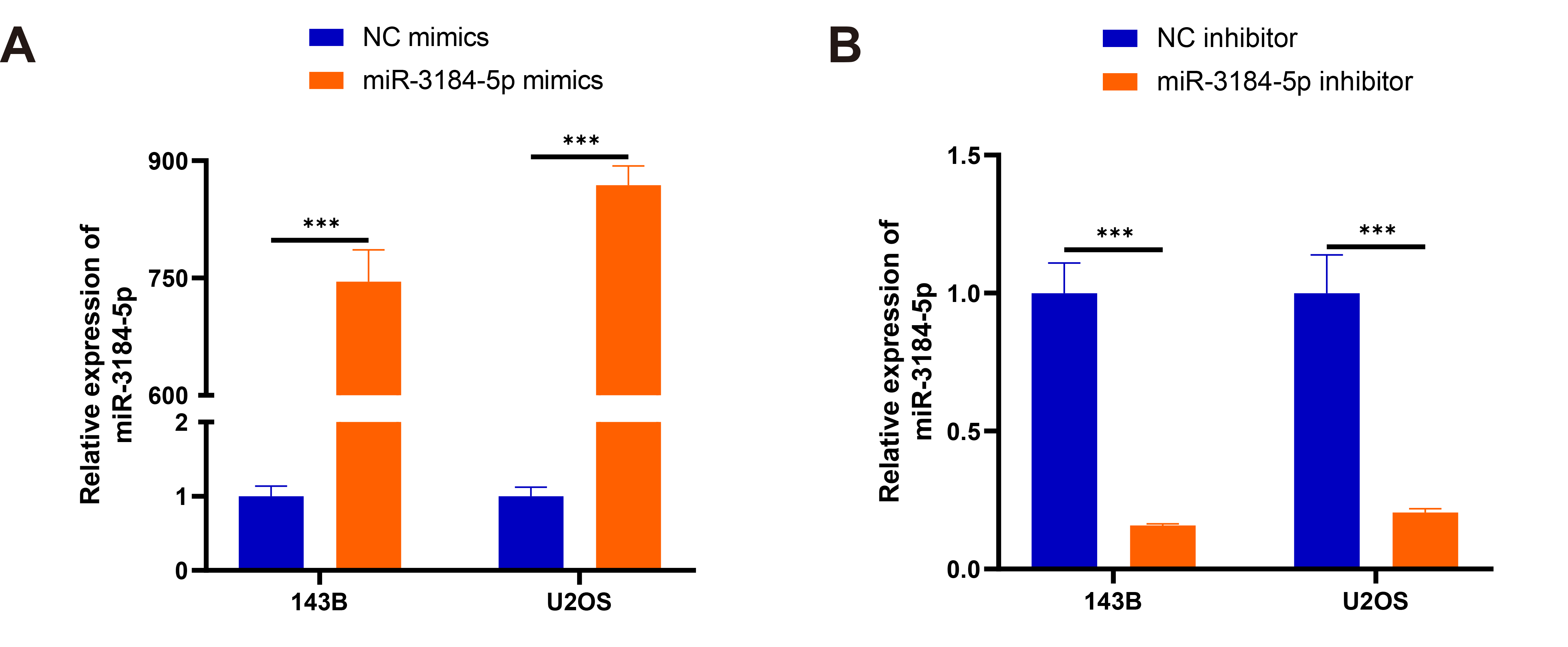

Supplement: Supplementary file 2 — Additional file 2: Fig. S1. Verification of transfection efficiency of miR-3184-5p in OSA cells. (A) After the transfection of mimic and inhibitor of miR-3184-5p, qRT-PCR was used to detect the transfection efficiencies. Data are expressed as mean ± SD (n = 3) (*, p < 0.05; **, p < 0.01; ***, p < 0.001). [file 40364_2022_442_MOESM2_ESM.tif]

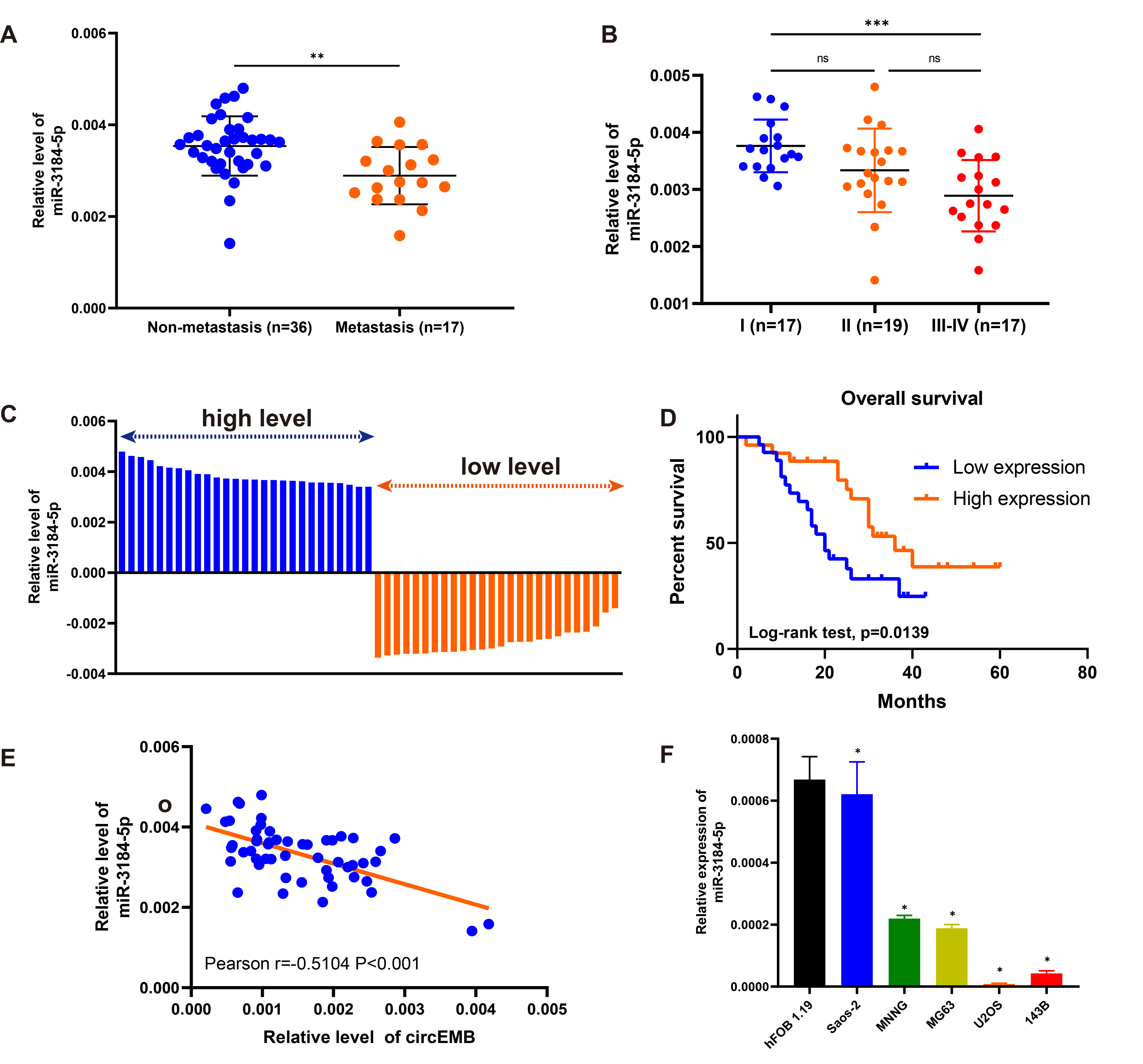

Supplement: Supplementary file 3 — Additional file 3: Fig. S2. The relationship between the miR-3184-5p and clinicopathological characteristics. (A) Comparison of miR-3184-5p expression between non-metastatic and metastatic OSA tissues. (B) Distribution characteristics of miR-3184-5p expression in the TNM stage of OSA. (C) In the 53-patients cohort, according to the median value of miR-3184-5p expression, they were divided into high expression group and low expression group. (D) Overall survival analysis of patients with low and high expression of miR-3184-5p using log-rank test and Kaplan–Meier analysis. (E) Correlation analysis between circEMB and miR-3184-5p in OSA tissues. (F) Relative miR-3184-5p levels in OSA cell lines and normal osteoblasts were ascertained by qRT-PCR. Data are expressed as mean ± SD (n = 3) (*, p < 0.05; **, p < 0.01; ***, p < 0.001). [file 40364_2022_442_MOESM3_ESM.tif]

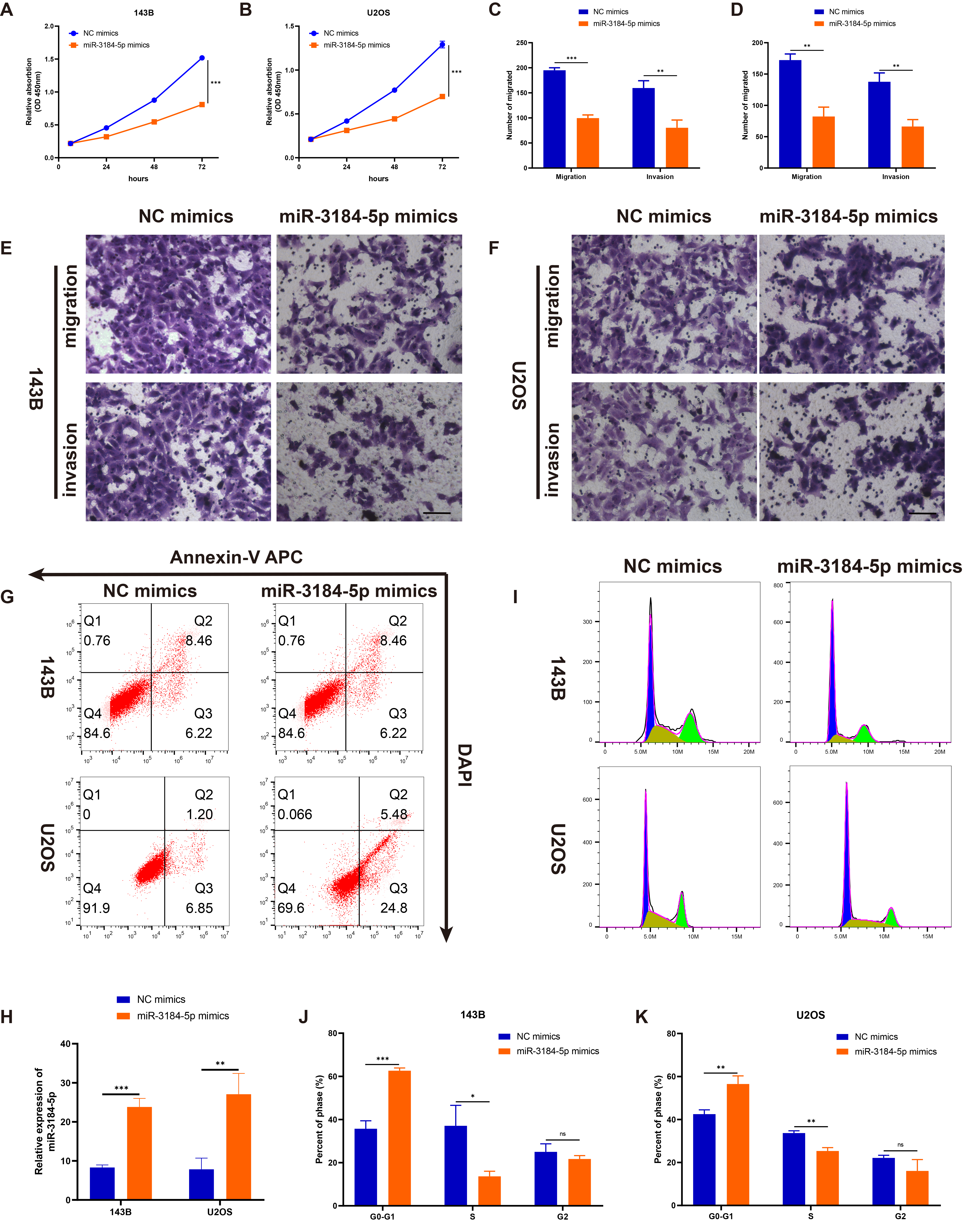

Supplement: Supplementary file 4 — Additional file 4: Fig. S3. Overexpression of miR-3184-5p was associated with decreased OSA cell migration and invasion, nor increased apoptosis and G1/S phase arrest. (A-B) The proliferation ability of OSA cells after overexpression of miR-3184-5p was determined by CCK-8. (C-F) Transwell assays detected the changes of invasion and metastasis ability of OSA cells with or without miR-3184-5p overexpression. (C and E) Refer to 143B, while (D and F) refer to U2OS. Scale bar, 100 μm. (G-H) Flow cytometric apoptosis analysis detected the changes of apoptosis of OSA cells with or without miR-3184-5p overexpression. (I-K) Flow cytometry assay showed the regulation of cell cycle by overexpressing miR-3184-5p. Data are expressed as mean ± SD (n = 3) (*, p < 0.05; **, p < 0.01; ***, p < 0.001). [file 40364_2022_442_MOESM4_ESM.tif]
